# Forward genetic analysis of monensin and diclazuril resistance in Eimeria tenella

**DOI:** 10.1016/j.ijpddr.2023.05.002

**Published:** 2023-05-24

**Authors:** Hongtao Zhang, Lei Zhang, Ganglin Ren, Hongbin Si, Xingju Song, Xianyong Liu, Xun Suo, Dandan Hu

**Affiliations:** a College of Animal Science and Technology, Guangxi University, China; b Guangxi Zhuang Autonomous Region Engineering Research Center of Veterinary Biologics, China; c Guangxi Key Laboratory of Animal Breeding, Disease Control and Prevention, Nanning, 530004, China; d Key Laboratory of Animal Epidemiology and Zoonosis of Ministry of Agriculture, National Animal Protozoa Laboratory, College of Veterinary Medicine, China Agricultural University, Beijing, China

**Keywords:** *Eimeria*, WGS, Monensin, Diclazuril, Linkage group selection

## Abstract

Worldwide distributed coccidiosis is caused by infection of both *Eimeria* species and *Cystoisospora* in the host intestine and causes huge economic losses to the livestock industry, especially the poultry industry. The control of such diseases relies mainly on chemoprophylaxis with anticoccidials, which has led to a very common drug resistance in this field. However, the genetic mechanisms underlying resistance to many anticoccidial drugs remain unknown. In this study, strains of *E. tenella* resistant to 250 mg/kg monensin were generated and characterized. Forward genetic approaches based on pooled genome sequencing, including experimental evolution and linkage group selection, were used to locate candidate targets responsible for resistance to monensin and diclazuril in *E. tenella*. A total of 16 nonsynonymous mutants in protein-coding genes were identified in monensin-resistant strains, and two genomic regions with strong selection signals were also detected in diclazuril-resistant strains. Our study reveals the genetic characterization of the experimental evolution and linkage group selection in *Eimeria* species, and also provides important information that contributes to the understanding of the molecular mechanism of drug resistance in coccidia.

## Highlights

• A monensin-resistant *E. tenella* line was generated and characterized.
• 16 nonsynonymous mutants were identified in monensin-resistant strains.
• Two genomic regions with strong selection signals were detected in diclazuril-resistant strains.

## Graphical abstract

**Figure:**
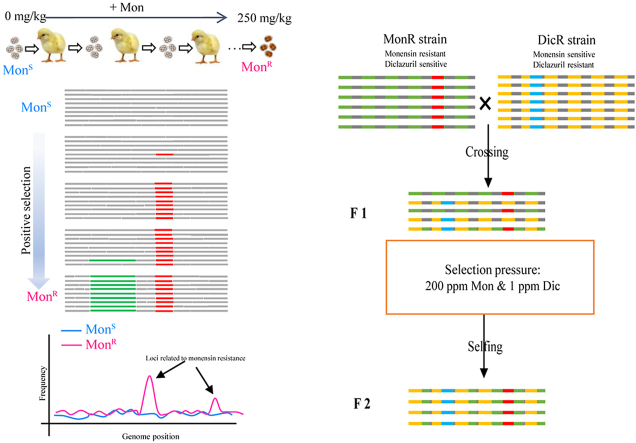

## 1 Introduction

Coccidian parasites, in the phylum of the intracellular protozoan Apicomplexa, are the major causes of coccidiosis in chickens, resulting in hematochezia, poor feed conversion ratios and chick mortality (Chapman et al., 2013). Coccidiosis occurs on almost all poultry farms worldwide, incurring costs of more than £10 billion to poultry production annually and compromising animal welfare (Williams, 1999; Blake et al., 2020).

Current control of coccidiosis in poultry is based primarily on chemoprophylaxis. Anticoccidial drugs were used in almost all commercial broiler farms before 2000 (Chapman and Jeffers, 2014). With pressure from customers and legislators, anticoccidial drugs continue to reduced usage in Europe and the USA (Shirley et al., 2007), and the abuse of anticoccidial drugs decreased in these regions. However, this situation is still common in many other countries. Due to the limited drugs available on the market and extensive use as a feed additive, anticoccidial drug resistance is pandemic in field *Eimeria* populations (Yadav and Gupta, 2001; Abbas et al., 2011; Zhang et al., 2013; Ojimelukwe et al., 2018). Unfortunately, despite common resistance to anticoccidials, the specific targets of these drugs and the genetic locus responsible for resistance remain unclear (Stock et al., 2018).

Forward genetic strategies have been widely adapted to discover the genetic basis of various phenotypes in apicomplexan parasites (Ariey et al., 2014; Behnke et al., 2016). Following the common application of next-generation sequencing (NGS), the resolution for genetic mapping improves dramatically as the high density of SNP markers in the genome compare to previously used markers, such as restriction fragment length polymorphism (RFLP), amplified fragment length polymorphism (AFLP), random amplified polymorphic DNA (RAPD) and minisatellite DNA. Experimental evolution and re-sequencing-based methodologies have been successfully used to identify molecular markers for artemisinin-resistant *Plasmodium* (Ariey et al., 2014) and *Toxoplasma* (Rosenberg et al., 2019); classical genetic linkage mappings have been performed to identify key virulence factors in *T. gondii* by crossing with different genotypes of parasites (Saeij et al., 2006; Behnke et al., 2011). In the case of *Eimeria* species, it parasitizes a single host and undergoes both asexual and sexual development during its life-cycle, and genetic crossing could effectively occur at the gamogony stage between different strains (Liu et al., 2022). On the other hand, it is inefficient to isolate single clones for the crossed progeny and also laborious to perform phenotypic analysis of recombinant clones. This is mainly due to the lack of a constitutive *in vitro* culture system (Shi et al., 2008; Bussière et al., 2018) and the need for several independent spaces for animals. Therefore, linkage group selection (LGS) based on pooled-seq is a much better solution for mapping causative loci for eukaryotic microbes that under natural/artificial selection (Culleton et al., 2005; Carter et al., 2007; Abkallo et al., 2017). More excitedly, Blake et al. (2011)(Blake et al., 2011) successfully mapped strain-specific immunity-related loci in *E. maxima* by using the LGS strategy together with AFLP markers.

In this study, we aimed to map drug-resistant loci in *Eimeria* by using forward genetics and pooled-seq. We generated a monensin-resistant *E. tenella* line by constitutive drug induction and analyzed selected genome regions. Linkage group selection was also performed by crossing the monensin-resistant strain to a diclazuril-resistant strain, and then identifying the two genomic regions responsible for monensin and diclazuril. Our study will provide basic knowledge on the molecular mechanism of drug resistance in coccidia.

## 2 Materials and methods

### 2.1 Ethical statement

The use of animals in this study was performed in accordance with the Institutional Animal Care and Use Committee guidelines and approved by the Administration Committee of Laboratory Animals in Guangxi University (approval number: Gxu-2021-013).

### 2.2 Animals

Two-to four-week-old San Huang broilers (Fufeng Animal Husbandry Co. LTD, Nanning, China) were used for passaging and drug induction. All birds were fed a coccidia-free diet and water *ad libitum*.

### 2.3 Parasites and development of resistance

The monensin-resistant Houghton strain (Mon^R^) was generated as previously described (Zhang et al., 2022). Briefly, sporulated oocysts of *E. tenella* Houghton strain (Mon^S^) were inoculated onto two-week-old chickens (5 × 10^4^/bird), and they were fed with gradient monensin (from 50 mg/kg to 300 mg/kg, MedChemExpress, Shanghai, China) two days before the infection and until the end of the passage. The concentration of monensin was elevated during the different generations of the passages depending on the total oocyst production per bird. The diclazuril-resistant strain (Dic^R^) was a gift from Prof. Xun Suo's laboratory. Dic^R^ is a field strain isolated from Henan province, China. It was evaluated to be resistant to 100 mg/kg diclazuril (Aladdin Biochemical Technology Co., Ltd., Shanghai, China) and sensitive to 1 mg/kg monensin (data not shown). The procedures for collection, purification and sporulation of the parasite were carried out as previously described (Eckert et al., 1995).

### 2.4 Genetic crosses

Ten chickens (4 weeks old) were infected orally with 5 × 10^3^ fresh oocysts of Mon^R^ strain and 5 × 10^3^ of Dic^R^ strain, and the mixed F1 cross progenies and self-cross progenies (major parts) of parent strains were collected from the feces during 5–9 dpi. Then, oocysts from the F2 cross progeny (5 × 10^4^/bird) were produced by inoculating the mixed F1 oocysts to another 10 chickens under double selection of 1 mg/kg diclazuril and 200 mg/kg monensin. The resulting F2 oocysts were the recombinant progeny of the Mon^R^ and Dic^R^ strains, and resistant to both drugs. This experiment was performed with two replicates.

### 2.5 Evaluation of drug susceptibility

The drug resistance of Mon^R^ and Mon^S^ to monensin was evaluated using different indexes. Five 2-week-old broilers were infected with fresh oocysts (1 × 10^4^/bird) for each strain and fed a diet with or without 200 mg/kg monensin. All feces were collected between 4- and 12-days post-infection (dpi) for counting total oocyst production using advanced McMaster counter (Haug et al., 2006). To measure body weight gain, chickens were weighed on days 0 and 12 dpi; for intestinal lesion scoring, chickens were sacrificed at 7 dpi, and ceca were removed for lesion scoring. Then, relative oocyst production (ROP), reduction of lesion scores (RLS), percentage optimum anticoccidial activity (POAA) and anticoccidial indexes (ACI) were calculated as described previously (Wang et al., 2006).

### 2.6 Merozoites purification and DNA extraction

Merozoites from Mon^R^, Mon^S^ and Dic^R^ strains, as well as crossed F1 and F2 populations, were collected as reported by Schwarz et al. (2010) with modifications. Briefly, 4-week-old broilers (n = 5) were orally inoculated with 5 × 10^5^ sporulated oocysts for each *E. tenella* strain/population, then the chickens were sacrificed for the cecum at 120 h post infection. The sheared cecum was digested (0.50% sodium taurodeoxycholate hydrate and 0.25% trypsin in PBS) at 42 °C for 30 min, then filtered through gauze and centrifuged to obtain a sediment containing impure merozoites, which were further filtered to obtain clean merozoites.

To isolate genomic DNA from each sample, purified merozoites were resuspended in 500 μL of CTAB (hexadecyltrimethylammonium bromide) lysis buffer (Solarbio, Beijing, China) and 0.4 mg/mL proteinase K (Solarbio, Beijing, China), and then lysed at 55 °C for 2 h. RNA was removed by incubation with 0.2 mg/mL RNase A (Solarbio, Beijing, China) at 37 °C for 30 min. Then, DNA was isolated following the classical phenol-chloroform DNA extraction protocol as described previously (Blake et al., 2003).

### 2.7 Library preparation and sequencing

Genomic DNA from each sample was sheared into ∼300 bp fragments and sequencing libraries were constructed using the VAHTS Universal DNA Library Prep Kit for Illumina V2 (Vazyme Biotech Co., Ltd, Nanjing, China) following the manufacturer's instructions. The qualified libraries were then sequenced using the 150 bp paired-end Illumina Novaseq 6000 platform according to the manufacturer's protocol. The resulting paired-end reads were uploaded to NCBI *via* SRA accession: PRJNA921877: https://www.ncbi.nlm.nih.gov/sra/PRJNA921877.

### 2.8 SNP calling

The paired-end short reads were trimmed and filtered by fastp (Chen et al., 2018), then qualified reads were aligned to the *E. tenella* reference genome (ToxoDB release 60) by BWA-mem (Li and Durbin, 2009) and the output SAM files were converted to BAM files using Samtools (Li et al., 2009). All aligned samples were then sorted and potential PCR duplicates were marked using the MarkDuplicates tool in GATK V4.0 (McKenna et al., 2010). Subsequently, gvcf files were generated by HaplotypeCaller in GATK and variants were called by the GenotypeGVCFs module after combining all gvcf files. The raw variants were then filtered by GATK with the parameters: QUAL <60.0, QD < 20.0, FS > 13.0, MQ < 30.0, MQRankSum < −1.65, ReadPosRankSum < −1.65. Finally, SNPs were obtained after filtering InDels by VCFtools (Danecek et al., 2011).

### 2.9 Genetic mapping and linkage analysis

For mapping of selected genomic regions after monensin induction or linkage group selections, SNPs from Mon^R^ and Mon^S^ or F2 and F1 offspring were selected to calculate the 5th power of the Euclidian distance (ED^5^) (Hill et al., 2013; Sun et al., 2018) and the ΔSNP-index (Takagi et al., 2013) by in-house R scripts based on previously published methods. The calculation of ED^5^ is based on the formula below:

$${ED}^{5}=\sqrt[{\frac{2}{5}}]{{\left(Aaa-Aab\right)}^{2}+{\left(Caa-Cab\right)}^{2}+{\left(Gaa-Gab\right)}^{2}+{\left(Taa-Tab\right)}^{2}}$$

where A_aa_, C_aa_, G_aa_ and T_aa_ represent the frequencies of bases A, C, G and T in the resistant (or F2) population, respectively. A_ab_, C_ab_, G_ab_ and T_ab_ represent the frequencies of bases A, C, G and T in the sensitive (or F1) population, respectively.

The SNP-index represents the allele frequency of an SNP that differs from the reference sequence. The ΔSNP-index for each locus (or region) was calculated by subtracting the SNP-index of the sensitive (or F1) population from that of the resistant (or F2) population. To reduce noise, SNPs with a sequencing depth ranging from 20 to 200 were involved. The ΔSNP-index was also calculated in 20 kb sliding windows with a step size of 5 kb using in-house R scripts. All codes are available upon request.

### 2.10 Statistics

The unpaired student's *t*-test was used to analyze total oocyst output, lesion score and average body weight gain. All bar plots depict the mean and standard deviations are shown as error bars.

## 3 Results

### 3.1 Induction of monensin-resistant *E. tenella* strains and phenotyping

**Fig. 1:**
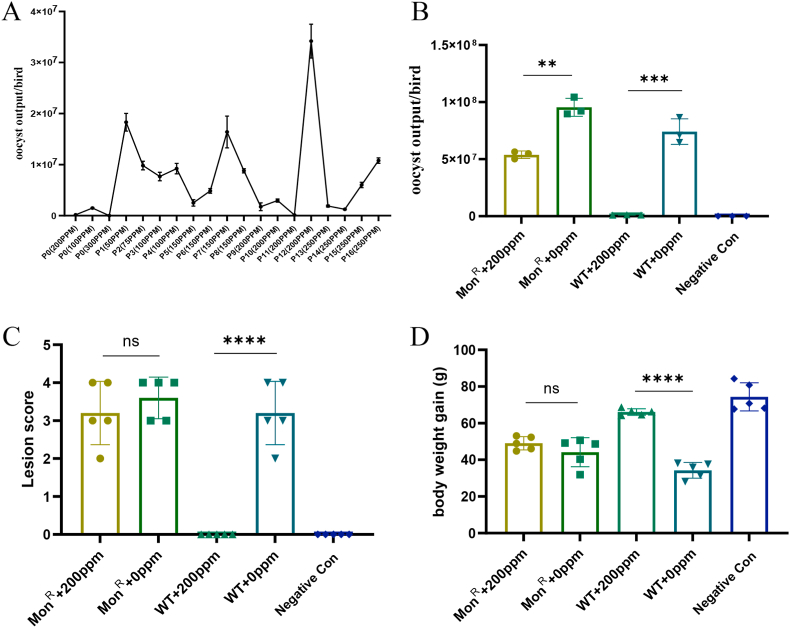
**Induction and phenotyping of monensin-resistant *Eimeria tenella* strain.** A: Induction of a monensin-resistant *E. tenella* strain (Mon^R^). Sporulated oocysts of the *E. tenella* Mon^S^ strain (5 × 10^4^/bird) were inoculated into two-week-old chickens and they were treated with different doses of monensin (from 50 mg/kg to 300 mg/kg). Oocyst output per bird was examined in each passage. To evaluate the susceptibility of Mon^R^ and Mon^S^ strains, five two-week-old broilers were infected with fresh oocysts (1 × 10^4^/bird) and treated with 0 mg/kg or 200 mg/kg monensin. Determination of oocyst output (B), intestinal lesion score of ceca (C) and average body weight gain in these groups. The uninfected and untreated groups served as negative control. ns: not significant; **: *P* < 0.01; ***: *P* < 0.001; ****: *P* < 0.0001.

To generate a monensin-resistant *E. tenella* strain, a sensitive *E. tenella* H strain (Mon^S^) was constitutively passaged in chickens under drug selection pressure. A small number of oocysts were detected after treatment with 100 mg/kg and 200 mg/kg monensin, called “leaky”, which usually occurs in the ionophores. With the 300 mg/kg monensin treatment, it completely prevented the production of oocysts. After 16 generations of selection, we obtained a Mon^R^ line that was resistant to 250 mg/kg monensin (Fig. 1A). Treatment with 200 mg/kg partially inhibited oocyst production of Mon^R^, which showed a relative oocyst production (ROP) rate of 56.4%, much higher than the ROP of the Mon^S^ strain (1.40%, Fig. 1B). The intestinal lesion caused by Mon^S^ infection in chickens was fully blocked by the 200 mg/kg monensin treatment with a 100% reduction of lesion score (RLS), but not significantly between the Mon^R^-infected groups with an RLS of only 11.1% (Fig. 1C). The body weight gain was significantly reduced by infection of both resistant and sensitive strains, and rescued significantly by monensin treatment in the Mon^S^ group, but not in the Mon^R^-infected group. The anticoccidial index (ACI) was 183.9 for Mon^S^ and 113.9 (<160) for the Mon^R^ strain; the percentage optimum anticoccidial activity (POAA) was calculated to be 66.1% (>50%) and 12.1% (<50%) for the Mon^S^ and Mon^R^ strains, respectively. These results indicate that Mon^S^ is a sensitive strain, while the Mon^R^ strain is totally resistant to 200 mg/kg monensin.

### 3.2 Identification of monensin-resistant mutants

**Fig. 2:**
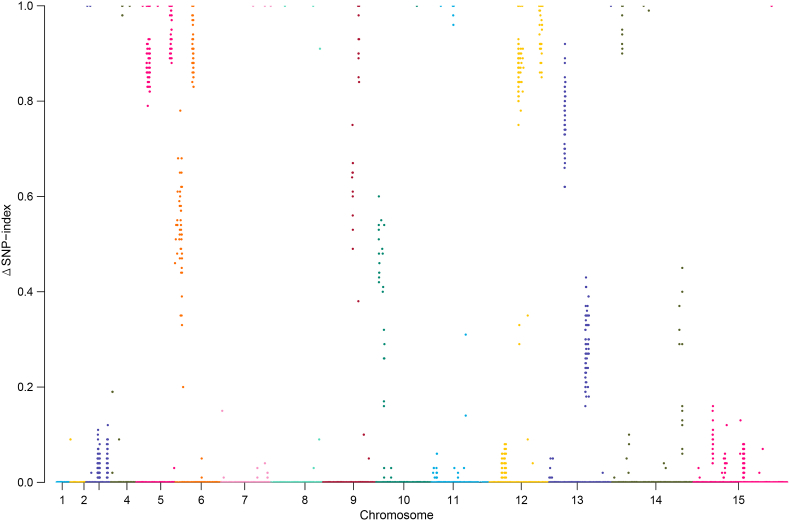
**Allele frequency changes in *E. tenella* after monensin induction as revealed by ΔSNP-index.** SNPs were called from the monensin-resistant Mon^R^ strain and its sensitive parent Mon^S^ strain, and the ΔSNP-index for each locus was calculated and plotted as a Manhattan plot. ΔSNP-index = SNP-index _(MonR)_ - SNP-index _(MonS)_.

**Fig. 3:**
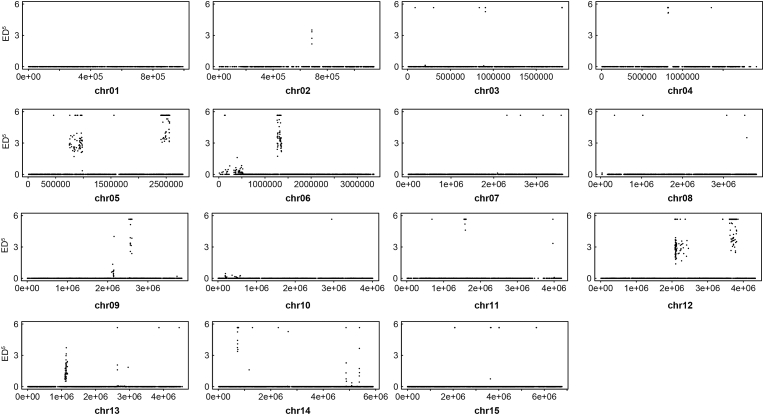
**Allele frequency changes in *E. tenella* after monensin induction as revealed by the Euclidian distance.** SNPs were called from the monensin-resistant Mon^R^ strain and its sensitive parent Mon^S^ strain, and the 5th power of the Euclidian distance (ED^5^) for each locus was calculated and plotted as a dot plot. The calculation methods for ED^5^ can be seen in the Materials and Methods section. SNPs with a higher ED^5^ value represent a greater allele frequency difference between sensitive and resistant strains.

To discover the genetic locus associated with monensin resistance in *Eimeria*, a sensitive *E. tenella* strain was produced under monensin selection for several generations. This is similar to the concept of experimental evolution, during which genetic regions associated with the phenotype are potently selected and exhibit higher allele frequencies. To analyze the genetic changes after constitutive monensin selection, genomic DNA of Mon^S^ and Mon^R^ strains was isolated and subjected to whole-genome resequencing. The obtained paired-end reads were mapped to the latest published reference genome with approximately 100-fold coverage, and then SNPs were called out for analysis. The allele frequency change was evaluated by calculating the ΔSNP-index and the 5th power of the Euclidian distance (ED^5^) between the Mon^S^ and Mon^R^ strains. We found that 6 major genomic regions in 5 chromosomes (chr05: 2401911–2557069; chr06: 1264321–1347392; chr09: 2528899–2602099; chr12: 2087267–2268816; chr12: 3627001–3811958; chr13: 1123575–1169509) showed a relatively high ΔSNP-index (Fig. 2) and ED^5^ (Fig. 3). Candidate SNPs informed by the ΔSNP-index and ED^5^ algorithms are listed in Supplementary dataset 1 and 2.

### 3.3 Linkage group selection revealing genomic regions related to monensin resistance

Since the likelihood of fusion of macro- and micro-gametocytes developed from different schizonts was rare in *Eimeria* species, most of the output oocysts were self-crossed offspring (Shirley and Harvey, 1996; Liu et al., 2022). It will be difficult to isolate a single oocyst from recombinant F1 progeny and also oocysts representing all genotypes after F1 self-crossing. Therefore, it seems pretty difficult to locate QTLs in *Eimeria* by means of linkage mapping. Alternatively, we intended using linkage group selection (LGS) analysis to find the monensin-resistant targets.

In this study, to further narrow down the candidate regions responsible for monensin resistance, the Mon^R^ strain was crossed with the Dic^R^ strain and recombinant F2 progeny were selected using 200 mg/kg monensin and 1 mg/kg diclazuril (Supplementary Fig. 1). SNPs of both parent strains and their F1 and F2 populations were called out for analysis. We found that the average allele frequency (0.045) in the Mon^R^ strain was very low, while the average allele frequency (0.976) in the Dic^R^ strain (Supplementary Fig. 2) was high. This can be explained by the *E. tenella* H strain origin of the Mon^R^ strain and the reference strain. However, even after infecting an equal number of oocysts, we obtained <0.001 allele frequencies for both replicates of the F1 population and identified moderate frequencies for the F2 population (0.644 and 0.645) (Supplementary Fig. 2). This result possibly indicates that the Dic^R^ strain has a fitness defect when co-infected with the Mon^R^ strain in the absence of selection pressure from anticoccidials.

**Fig. 4:**
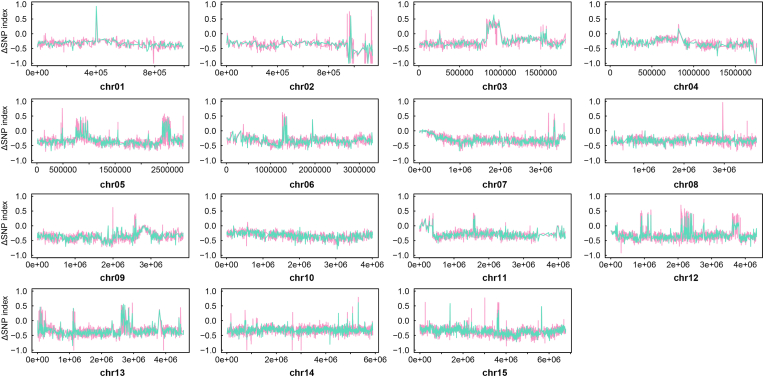
**Identification of selected genomic regions after monensin induction by linkage group selection.** Genetic cross was performed by infecting 10 four-week-old chickens with 5 × 10^3^/bird Mon^R^ strain and Dic^R^ strain. Recombinant F2 oocysts were harvested from infections of mixed F1 oocysts under a selection of 1 mg/kg diclazuril and 200 mg/kg monensin. The genomes of the parent and offspring populations were then sequenced, and SNPs were called and filtered for analysis. Monensin-related genomic regions were determined by the ΔSNP-index in a 20 kb sliding window, with a 5 kb step between the F2 population and the monensin-sensitive Dic^R^ parent strain. The red and green lines represent two independent crossing experiments. (For interpretation of the references to colour in this figure legend, the reader is referred to the Web version of this article.)

To find SNPs associated with monensin resistance, irrelevant loci in the F2 population were removed by calculating the ΔSNP-index between the F2 population and the Dic^R^ strain. This resulted in the selection of SNPs in the F2 population from 8 major genomic regions in 5 chromosomes and many other single SNPs inherited from the Mon^R^ strain (Fig. 4). After annotating and filtering the candidate regions based on our experimental evolution analysis, we found common missense variants to be located in 16 protein-coding regions and a Gln-transfer RNA (Table 1). The Gln to Arg alteration occurs in the DEXDc domain of an ATP-dependent RNA helicase (ETH2_0534200), but the exact change in its function should be further validated.

**Table 1:** Candidate genes with missense variants for monensin resistance.

Gene ID	AF-S	AF-R	Δ	ED^5^	LGS1	LGS2	variant type	DNA	Protein	Product Description
ETH2_1259200	0	0.97	0.97	5.66	0.35	0.28	missense	22998C > G	His7666Gln	type I fatty acid synthase, putative
ETH2_1258300	0	1	1	5.66	0.33	0.41	missense	275G > A	Ser92Asn	microneme protein, putative
ETH2_1257900	0	1	1	5.66	0.3	0.27	missense	2956A > G	Thr986Ala	Myb-like DNA-binding domain containing protein, putative
ETH2_1257100	0	0.98	0.98	5.66	0.42	0.24	missense	19G > A	Ala7Thr	hypothetical protein
ETH2_1236200	0.21	1	0.79	1.67	0.5	0.4	missense	545C > T	Ala182Val	sushi domain-containing protein/SCR repeat-containing protein, putative
ETH2_1234400	0.10	0.99	0.88	3.31	0.58	0.27	missense	1556A > C	His519Pro	hypothetical protein, conserved
ETH2_1231600	0.16	1	0.84	2.31	0.7	0.59	missense	1712C > G	Ala571Gly	PPR repeat-containing protein, putative
ETH2_0940000	0	0.98	0.98	5.66	0.37	0.39	missense	56A > G	Gln19Arg	Gln transfer RNA (ctg)
ETH2_0624800	0.08	1	0.92	3.66	0.53	0.56	missense	2059C > A	Leu687Ile	hypothetical protein, conserved
ETH2_0535200	0	0.97	0.97	5.66	0.31	0.27	missense	922A > G	Ser308Gly	COG3639: ABC-type phosphate/phosphonate transport system, permease component, related
ETH2_0535000	0	0.98	0.98	5.66	0.33	0.32	missense	887T > C	Ile296Thr	PT repeat family protein, related
ETH2_0534800	0	1	1	5.66	0.44	0.3	missense	1655T > G	Val552Gly	mRNA processing protein, putative
ETH2_0534500	0	0.98	0.98	5.66	0.25	0.21	missense	3157T > C	Ser1053Pro	hypothetical protein, conserved
ETH2_0534200	0	0.98	0.98	5.66	0.12	0.29	missense	1307A > G	Gln436Arg	ATP-dependent RNA helicase, putative
ETH2_0508800	0.08	1	0.91	3.59	0.24	0.32	missense	2444C > T	Thr815Ile	pseudouridylate synthase 1, putative
ETH2_0508100	0.097	1	0.90	3.39	0.21	0.33	missense	1751A > G 239A > G	Glu584Gly Glu80Gly	ftsJ-like methyltransferase domain-containing protein, putative

AF-S: allele frequency of Mon^S^; AF-R: allele frequency of Mon^R^; Δ: ΔSNP-index between Mon^R^ and Mon^S^; LGS1/LGS2: ΔSNP-index between SNP of F2 and Dic^R^ in the two linkage group selection experiments.

### 3.4 Identification of candidate genomic regions for diclazuril resistance

**Fig. 5:**
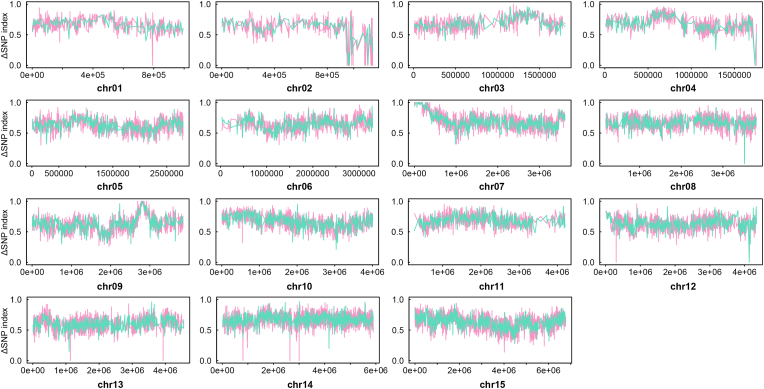
**Identification of selected genomic regions for diclazuril resistance by linkage group selection.** Genetic cross was performed by infecting 10 four-week-old chickens with 5 × 10^3^/bird Mon^R^ strain and Dic^R^ strain. Recombinant F2 oocysts were harvested from infections of mixed F1 oocysts under a selection of 1 mg/kg diclazuril and 200 mg/kg monensin. The genomes of the parent and offspring populations were then sequenced, and SNPs were called and filtered for analysis. Diclazuril-related genomic regions were determined by the ΔSNP-index in a 20 kb sliding window, with a 5 kb step between the F2 and F1 populations. The red and green lines represent two independent crossing experiments. (For interpretation of the references to colour in this figure legend, the reader is referred to the Web version of this article.)

Following treatment of sensitive individuals with lethal doses of anticoccidial drugs, two loci responsible for monensin and diclazuril resistance were selected in the F2 progeny. Thus, the diclazuril-related locus could also be detected based on allele frequency changes after drug selection (Supplementary Fig. 1). The ΔSNP-index between the F2 and F1 populations was used to reflect the allele frequency changes resulting from drug selection. Two genomic regions with a high ΔSNP-index were shown in both LGS replicates (Fig. 5). The two regions located at chr07: 9659–766820 and chr09: 2643130–2928871 contain 119 and 170 SNPs, respectively, with ΔSNP-index higher than 0.8. These SNPs caused 26 nonsynonymous mutants in protein-coding genes (Table 2), but the SNPs may also have effects on other genes located in these selected regions. We also listed the coding genes in the genomic region of chr09 (2702262–2875092) with ΔSNP-index >0.9 (Supplementary dataset 3), which may also be considered in further validation experiments.

**Table 2:** Candidate genes with missense variants for diclazuril resistance.

gene id	LGS 1	LGS 2	DNA	Protein	Product Description
ETH2_0700100	0.98	0.97	1748G > A	Arg583Gln	hypothetical protein, conserved
ETH2_0700700	0.98	1	40C > T	Arg14Cys	Methyltransferase domain containing protein, putative
ETH2_0700900	1	1	79T > A	Ser27Thr	myosin light chain TgMLC1, putative
ETH2_0701100	1	1	1027T > G 1300A > G	Ser343Ala Ile434Val	hypothetical protein, conserved
ETH2_0701300	1	1	6727T > G 17628T > A	Cys2243Gly Asp5876Glu	phosphatidylinositol 3-and 4-kinase domain-containing protein, putative
ETH2_0701400	1	1	2831T > C 1712C > A	Leu944Pro Ala571Glu	cation-transporting ATPase, putative
ETH2_0702400	0.88	0.96	4271C > A	Ser1424Tyr	RNA recognition motif-containing protein, putative
ETH2_0702500	0.95	0.89	918G > C	Arg306Ser	hypothetical protein, conserved
ETH2_0702600	0.83	0.81	114G > C 298A > G	Gln38His Thr100Ala	Eukaryotic aspartyl protease, putative
ETH2_0703000	0.97	0.91	2146C > G	Leu716Val	hypothetical protein, conserved
ETH2_0703100	0.94	0.96	2244A > C	Lys748Asn	transporter, putative
ETH2_0703500	0.94	0.89	4850T > C 2864T > C	Leu1617Pro Val955Ala	hypothetical protein, conserved
ETH2_0705300	0.83	0.89	5537A > G	Lys1846Arg	thrombospondin type 1 domain-containing protein, putative
ETH2_0708100	0.88	1	865T > G	Ser289Ala	hypothetical protein, conserved
ETH2_0710600	0.81	0.85	560T > C	Leu187Ser	translin, putative
ETH2_0942500	0.91	0.82	48A > C 54T > A	Lys16Asn Asn18Lys	hypothetical protein, conserved
ETH2_0944100	0.9	0.9	1468T > A	Cys490Ser	PPPDE putative peptidase domain containing protein, putative
ETH2_0944600	0.97	0.96	637A > G	Thr213Ala	hypothetical protein, conserved
ETH2_0945300	1	0.96	5657A > C	Gln1886Pro	hypothetical protein, conserved
ETH2_0945700	0.91	1	65G > A	Gly22Glu	hypothetical protein, conserved

LGS1/LGS2: ΔSNP-index between SNP of F2 and F1 in the two linkage group selection experiments.

## 4 Discussion

Coccidiosis remains economically important in the livestock industry and global food security. Common drug resistance to anticoccidials adds further difficulties to the control of this disease (Abbas et al., 2011). However, the genetic mechanisms of drug resistance to most anticoccidials remained unclear. Here, we used experimental evolution and linkage group selection along with pooled genome sequencing strategies to locate candidate genome regions/loci responsible for monensin and diclazuril resistance. Our results provide important information and direction for understanding the molecular mechanism of drug resistance in coccidia.

In this study, two *E. tenella* strains with different genetic backgrounds and different drug resistance were crossed and specific selection pressure was applied to the uncloned progeny, resulting in a crossed population with drug resistance to both anticoccidials. Genetically, different markers from the two parents would be randomly separated in equal proportions into their recombinant offspring (Supplementary Fig. 1). However, in our study, we obtained very low (<0.01) allele frequencies in the SNPs of the F1 generation. This can be explained by the likelihood of cross-over of macro- and micro-gametocytes developed from different parents. Liu et al. (2022) demonstrated that about 3–11% of the oocysts were recombinant offspring, while the rest of the oocysts were self-bred from each parent. Thus, different doses of infection or parents with different fitness will result in a biased or polarized distribution of SNPs in the unselected F1 population. After selection with both drugs, individuals not possessing the drug-resistant alleles of both drugs would be eliminated from the final population along with their susceptibility-conferring alleles (Culleton et al., 2005; Carter et al., 2007). As a result, the allele frequency of the causative locus in the final population (F2) would be high (even to be fixed), and the polymorphism or heterozygosity of the genome region around the causative locus could also be reduced due to the selective sweep (Wootton et al., 2002; Nair et al., 2003). Additionally, the average allele frequencies of the F2 populations after drug selection are again close to 1:1 (0.644/0.645) inherited from two parents.

Previous studies on *Toxoplasma gondii* by Arrizabalaga's group have shown that disruption of a mitochondrial MutS DNA repair enzyme, TgMSH-1, confers monensin resistant to the parasite (Garrison and Arrizabalaga, 2009). However, in our study, we could not find any SNP on the *Eimeria* ortholog of TgMSH-1. Actually, we attempted to directly knock out the ortholog of TgMSH-1 in *E. tenella* for validating its role in monensin resistance in *Eimeria*. We were successful in achieving transfectants (∼20% positive rate in its 3rd generation), but monensin treatment did not increase its positive rate (preliminary unpublished data). These results may suggest that different mechanisms of monensin resistance exist in *Toxoplasma* and *Eimeria*.

Forward genetic mapping strategies have been successfully used in *Eimeria* to locate gene loci responsible for strain-specific immunity (Blake et al., 2011) and drug-resistance. However, it still has drawbacks in this parasite. 1). It requires cloned strains with very clear genetic background, and the *Eimeria* species do not have a constitutive *in vitro* culture system (Shi et al., 2008; Bussière et al., 2018). Thus, laborious animal experiments were needed for the establishment of a cloned strain. 2). Experimental evolution of drug-resistant *Eimeria* also needs several generations of passages *in vivo*, which is time-consuming even it's better than in other asexual developed parasites (Rosenberg et al., 2019). 3). *Eimeria* has both sexual and asexual development, the spontaneous mutation rate of sexual development is much higher than asexual development (Lynch, 2010; Rosenberg et al., 2019). Compared to the asexual development, the high mutation rate of sexual development makes it faster to obtain drug-resistant strain, but also results in a lot of non-specific mutations.

Due to the high quality of the *E. tenella* reference genome (Reid et al., 2014; Aunin et al., 2021), genetic mapping can be successfully performed. However, there are still challenges in validating candidate mutants due to the large number of candidates and the limitations of genetic manipulation in *Eimeria*. We previously developed a CRISPR-Cas9-based gene editing tool for *Eimeria*, which could be successfully used to manipulate the *Eimeria* genome (Hu et al., 2020; Tang et al., 2020). However, the relatively low recombination rate limits its use for point mutations/replacement. Therefore, other strategies should be developed for *Eimeria* genome editing, such as knocking out or repressing key proteins responsible for non-homologous end-joining or enhancing the pathway of homologous recombination by small molecular compounds (Smirnikhina et al., 2019).

Collectively, we used two forward genetic methods to map candidate loci associated with monensin and diclazuril resistance. We provided several candidate genes for further experimental analysis and also revealed genetic characterization of experimental evolution and linkage group selection in *Eimeria* species. Such knowledge will contribute to dissecting the molecular mechanisms of drug resistance in coccidia.

## Author contributions

**Hongtao Zhang:** Investigation, Writing - original draft, Visualization; **Lei Zhang:** Investigation, Visualization; **Ganglin Ren:** Validation, Writing - original draft; **Hongbin Si**: Writing - review & editing; **Xingju Song:** Conceptualization, Supervision; **Xianyong Liu:** Writing - review & editing; **Xun Suo:** Conceptualization, Writing - review & editing; **Dandan Hu:** Software; Funding acquisition; Supervision. All authors read and approved the final manuscript.

## Funding

This work was supported by the Natural Science Foundation of Guangxi Zhuang Autonomous region (grant no. 2021GXNSFBA220057), the 10.13039/501100001809National Natural Science Foundation of China (grant no. 32102694), and the Specific Research Project of Guangxi for Research Base and Talents (grant no. AD21075028).

## Declaration of competing interest

The authors declare that the research was conducted in the absence of any commercial or financial relationships that could be construed as a potential conflict of interest.

## Appendix A Supplementary data

Supplementary figure 2 Allele frequencies of different populations in linkage group selection. Allele frequencies were calculated to be 50 kb sliding windows with 10 kb steps. Red, pink, green and blue lines represent Mon^R^ strain, Dic^R^ strain, F1 and F2 populations. Supplementary figure 2

Supplementary figure 1 Diagrams for the linkage group selection in *Eimeria*. A: Procedure for *E. tenella* crossing. Briefly, two *E. tenella* lines with different backgrounds and susceptibility to monensin and diclazuril were crossed by superinfection in the chicken and then the F1 population was generated without selection, with the F1 population containing recombinant (yellow) and non-recombinant oocysts. The F2 population was generated under selection pressure with lethal doses of both anticoccidials, and the remaining oocysts were recombinant progeny of the two parents. **B:** Genetic changes during LGS. Specific markers for the Mon^R^ and Dic^R^ strains are marked in green and yellow, respectively. Furthermore, the loci responsible for monensin and diclazuril resistance are shown in red and blue, respectively. In the F1 population, pooled-seq would result in mixed allele frequencies. After drug selection, only oocysts with both loci responsible for monensin and diclazuril resistance would survive, and the allele frequencies in the causative regions would be elevated or even fixed. Supplementary figure 1
