# Supplementary material for: Forward genetic analysis of monensin and diclazuril resistance in Eimeria tenella
Source: Int J Parasitol Drugs Drug Resist. 2023 May 24;22:44–51. doi: 10.1016/j.ijpddr.2023.05.002 (PMC10238932; doi:10.1016/j.ijpddr.2023.05.002)
Supplement: Supplementary figure 2 — Allele frequencies of different populations in linkage group selection. Allele frequencies were calculated to be 50 kb sliding windows with 10 kb steps. Red, pink, green and blue lines represent MonR strain, DicR strain, F1 and F2 populations. [file mmc1.pdf]

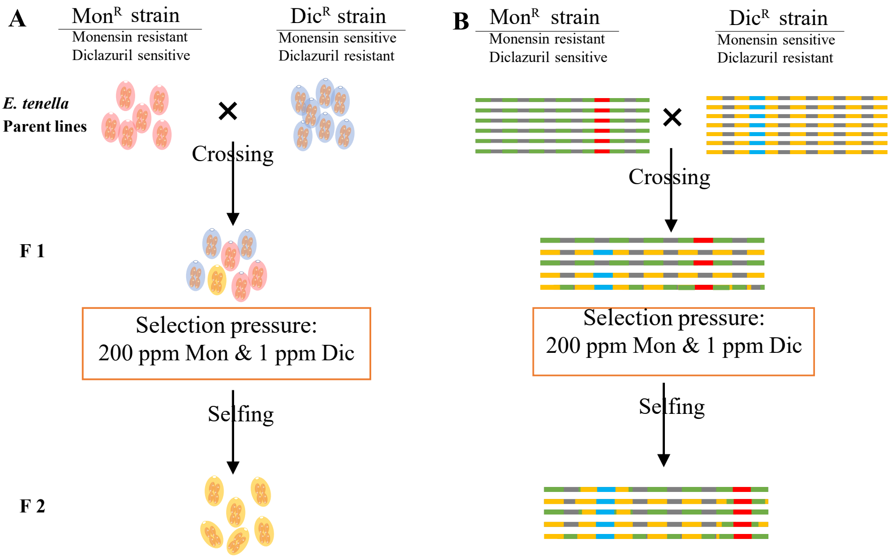
A
MonR strain
Monensin resistant
Diclazuril sensitive
DicR strain
Monensin sensitive
Diclazuril resistant
E. tenella
Parent lines
×
Crossing
F 1
Selection pressure:
200 ppm Mon & 1 ppm Dic
Selfing
F 2
B
MonR strain
Monensin resistant
Diclazuril sensitive
DicR strain
Monensin sensitive
Diclazuril resistant
×
Crossing
Selection pressure:
200 ppm Mon & 1 ppm Dic
Selfing
