# Supplementary figures and images for: Forward genetic analysis of monensin and diclazuril resistance in Eimeria tenella
Source: Int J Parasitol Drugs Drug Resist. 2023 May 24;22:44–51. doi: 10.1016/j.ijpddr.2023.05.002 (PMC10238932; doi:10.1016/j.ijpddr.2023.05.002)

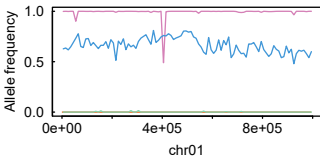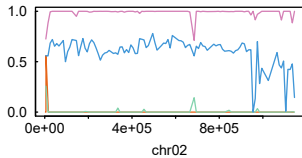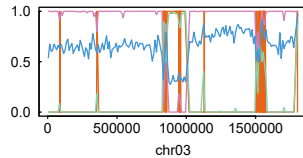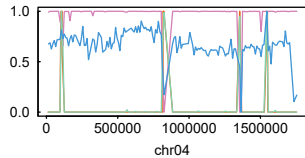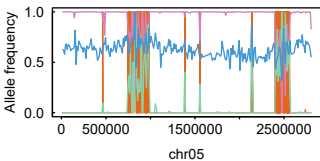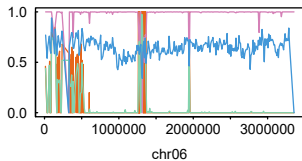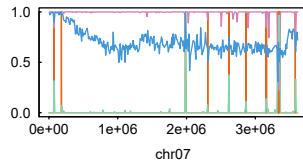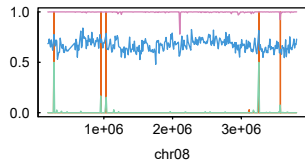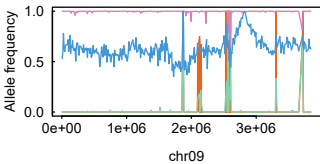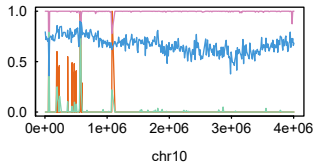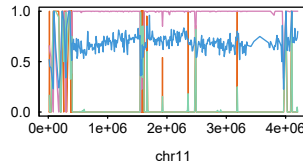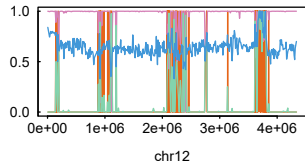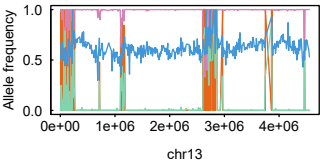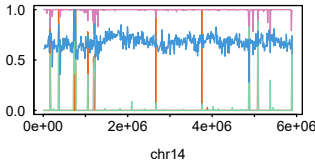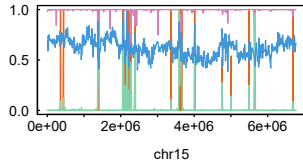

Supplement: Supplementary figure 1 — Diagrams for the linkage group selection in Eimeria. A: Procedure for E. tenella crossing. Briefly, two E. tenella lines with different backgrounds and susceptibility to monensin and diclazuril were crossed by superinfection in the chicken and then the F1 population was generated without selection, with the F1 population containing recombinant (yellow) and non-recombinant oocysts. The F2 population was generated under selection pressure with lethal doses of both anticoccidials, and the remaining oocysts were recombinant progeny of the two parents. B: Genetic changes during LGS. Specific markers for the MonR and DicR strains are marked in green and yellow, respectively. Furthermore, the loci responsible for monensin and diclazuril resistance are shown in red and blue, respectively. In the F1 population, pooled-seq would result in mixed allele frequencies. After drug selection, only oocysts with both loci responsible for monensin and diclazuril resistance would survive, and the allele frequencies in the causative regions would be elevated or even fixed. [file mmc2.pdf]
